# Importance of Cry Proteins in Biotechnology: Initially a Bioinsecticide, Now a Vaccine Adjuvant

**DOI:** 10.3390/life11100999

**Published:** 2021-09-23

**Authors:** Maria Cristina Gonzalez-Vazquez, Ruth Abril Vela-Sanchez, Norma Elena Rojas-Ruiz, Alejandro Carabarin-Lima

**Affiliations:** 1 Centro de Investigaciones en Ciencias Microbiologicas, Instituto de Ciencias, Benemerita Universidad Autonoma de Puebla, Puebla 72000, PU, Mexico; crispi333@yahoo.com.mx (M.C.G.-V.); normaelena_rojas@yahoo.com.mx (N.E.R.-R.); 2 Licenciatura en Biotecnología, Benemerita Universidad Autonoma de Puebla, Puebla 72000, PU, Mexico; ruth.vela1596@gmail.com

**Keywords:** *Bacillus thuringiensis*, Cry proteins, adjuvant

## Abstract

A hallmark of *Bacillus thuringiensis* bacteria is the formation of one or more parasporal crystal (Cry) proteins during sporulation. The toxicity of these proteins is highly specific to insect larvae, exerting lethal effects in different insect species but not in humans or other mammals. The aim of this review is to summarize previous findings on *Bacillus thuringiensis*, including the characteristics of the bacterium, its subsequent contribution to biotechnology as a bioinsecticide due to the presence of Cry proteins, and its potential application as an adjuvant. In several studies, Cry proteins have been administered together with specific antigens to immunize experimental animal models. The results have shown that these proteins can enhance immunogenicity by generating an adequate immune response capable of protecting the model against an experimental infectious challenge, whereas protection is decreased when the specific antigen is administered without the Cry protein. Therefore, based on previous results and the structural homology between Cry proteins, these molecules have arisen as potential adjuvants in the development of vaccines for both animals and humans. Finally, a model of the interaction of Cry proteins with different components of the immune response is proposed.

## 1. Introduction

The aim of this review is to show the potential use of Cry proteins as vaccine adjuvants. First, general information is presented about *Bacillus thuringiensis* (Bt), a bacterium that can form spores and produce Cry proteins, which are natural bioinsecticides. Then, the review focuses on a new property attributed to Cry proteins: immunopotentiators of the immune response or adjuvants. This is an innovative application to explore because, currently, few adjuvants are licensed for vaccine formulations, and several are in the research stage [1]. Additionally, identifying a natural adjuvant that does not cause harm to humans will be beneficial, and Cry proteins are optimal candidates to play this role in the near future [2,3,4]. Moreover, recent research in animal models has shown that Cry proteins are not toxic [5], and their use in human vaccines could be safe.

## 2. Bacillus Thuringiensis (Bt)

*B. thuringiensis* was isolated for the first time in 1902 by the Japanese scientist Ishiwata, who was studying the cause of mortality in silkworm larvae; thus, this disease was also called Soto disease. Ishiwata initially named this bacterium *Bacillus sotto* [6]. A few years later, in 1911, a German scientist, Ernst Berliner, isolated a bacterial strain in dead moth larvae in Mediterranean flour, located in a flour mill in the German state of Thuringia. For this reason, Ernst named this Bacillus *B. thuringiensis* (Bt) [7]. Subsequently, the probable mechanism of cytotoxic action of particular Bt inclusions, called parasporal, was shown in silkworm larvae (*Bombyx mori*). Changes in the permeability of the intestinal walls of the insect were observed, consequently causing its death. These results showed that the parasporal inclusions contained crystals of δ-endotoxin, which was the cause of the larva deaths [8,9]. The successful use of Bt in agriculture lies in the production of crystal proteins called Cry, which have specific cytotoxic activity against different insect orders, such as Lepidoptera, Diptera, Coleoptera, Hymenoptera, Homoptera, Orthoptera, and Mallophaga [10,11,12].

The first product based on Bt toxins was commercialized in 1938 in France for moth control. In 1958, Bt products became commercially available in the USA [13]. The predominant use of Bt toxins is for the control of agricultural pests, which is carried out by genetically modified plants (Bt plants) [14,15,16,17]. Several excellent reviews have been published on the control of pests using Cry toxins [18,19,20,21]. Table 1 shows some of the pests controlled by Cry toxins.

### 2.1. Overview of Bt and Spore Formation

Bt is a Gram-positive, catalase-positive, oxidase-negative, strictly aerobic bacterium with peritrichous flagella that enable motility. It is generally found in soils, but it is also present in water, insects, some grains, and the environment [11,22,23,24,25]. Bt has two life cycles: vegetative growth and a sporulation phase. One of the main characteristics of Bt that distinguishes it from other bacilli of the same genus is the intracellular presence of a protein crystal [11,15,17,26].

**Figure 1:**
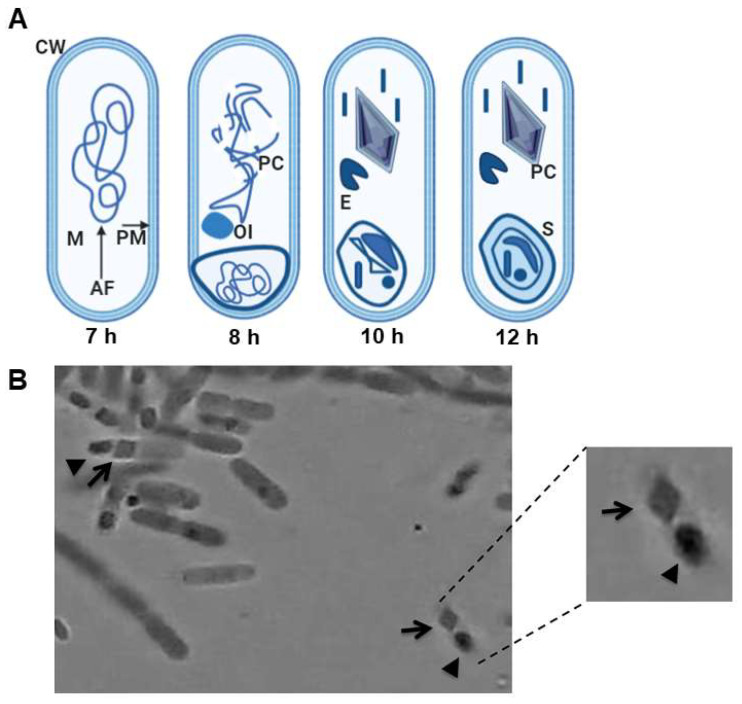
(**A**) An illustrative schematic diagram of sporulation of Bt over several hours. Mesosome (M), cell wall (CW), plasma membrane (PM), axial filament (AF), ovoid inclusion (OI), bipyramidal parasporal crystal (PC), exosporium (E), spore (S). Based on Bulla et al. [27]. (**B**) Microscopy of Bt at 100x. The inset shows a digitally enlarged Bt cell showing the bipyramidal parasporal crystal. The black arrows show the crystal (PC), and the arrowheads show the spore (S).

During the sporulation phase, Bt forms a protein crystal. The development of the spore and crystal comprises seven distinct stages: (a) phase I: axial filament formation; (b) phase II: formation of the forespore septum; (c) phase III: parasporal crystals and pre-spore formation; (d) phases IV–VI: exospore formation, primordial cell wall development, and the transformation of spore nucleoids; (e) phase VII: spore maturation and cell lysis (Figure 1) [6,10,27,28]. Crystals are synthesized after stage II of sporulation and accumulate in the cell where they can represent up to 30% of the dry weight of the sporulated cells [29,30]. Several authors have described the presence of different forms of Bt crystals, such as dipyramidal, pyramidal, cuboidal, flat rhomboid, spherical, and rectangular. The most commonly found shape is the dipyramidal crystal. These crystals may include one or more δ-endotoxins, also known as Cry proteins [31,32,33].

In 1981, the cry gene that encodes for the toxin protein in Bt was cloned for the first time [34]. To date, 731 genes encoding Cry proteins have been found with 272 holotypes [35]. Cry proteins are classified into 50 groups and several subgroups, depending on the host specificity, structure, and mechanism of action. These proteins have molecular weights between 30 kDa and 140 kDa [36,37].

### 2.2. Cry Proteins

In 1989, a nomenclature was proposed to classify proteins according to their sequence and specificity. In this initial nomenclature, there were only four classes. The first class included proteins with action against Lepidoptera with a size of approximately 130–140 kDa. The second class included smaller proteins (65 kDa) with activity against Lepidoptera and Diptera; this class included only two members: CryIIA and CryIIB. The third class constituted the active toxin against Coleoptera, CryIIIA. The last class was Cry1A, the members of which were closely related: they were called Cry1Aa, Cry1Ab, and Cry1Ac [14].

**Figure 2:**
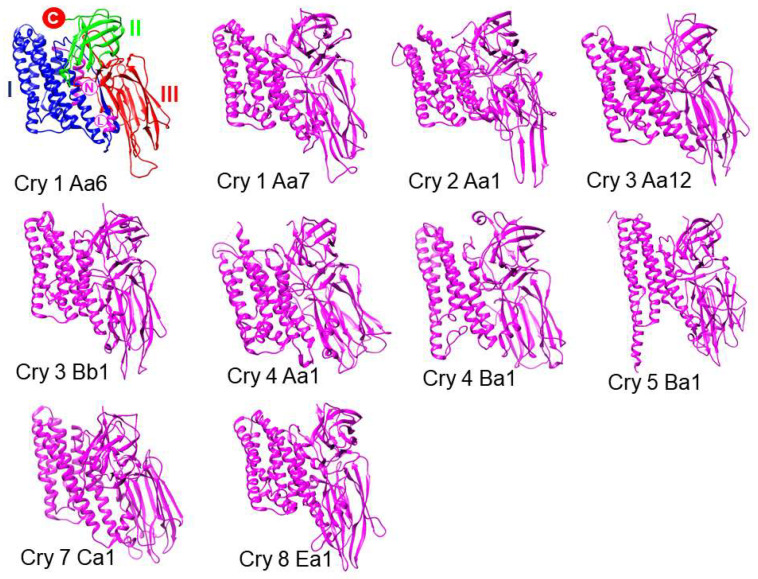
Structural representation of some crystallized Cry proteins (toxins). The Cry 1 Aa6 toxin Domains I-III are indicated in blue, green, and red, respectively, and the N- and C-termini are shown by circles filled with red and white backgrounds, respectively. The Linker connecting Domains I and II is shown by a circle filled with a white background with the letter in magenta. Modeling was performed in UCSF Chimera (https://www.rbvi.ucsf.edu/chimera (accessed on 18 September 2021)).

In 1998, a new nomenclature was published classifying toxins solely by their amino acid sequence. On this basis, most proteins are related and contain up to five conserved domains. Subsequently, a slightly modified name system was adopted in which each toxin receives a name that incorporates four levels. First, in general, toxins that share at least 45% identity in their sequence have the same number. The second rank (A) is used to distinguish sequences that share between 45% and 78% identity. Those that share between 78% and 95% identity are distinguished at the tertiary level (a). Finally, the quaternary range is used to identify certain differences. Subsequently, in 2003, Cry proteins were classified into three groups through a phylogenetic approach. The group with the majority of Cry toxins is known as the family of three domains since they contain three structural domains. This family contains the largest group of Cry proteins, which are globular molecules that contain three structural domains connected by simple bonds. A particular characteristic of the members of this family is the presence of protoxins with two different lengths. Long protoxins are approximately twice the length of most toxins. The C-terminal extension found in long protoxins is dispensable for toxicity and is believed to play a role in the formation of crystal inclusion bodies within the bacteria [14]. To date, the three dimensional structures of 12 Cry toxins without modifications (Cry1Aa6, Cry1Ac7, Cry1Ac8, Cry2Aa, Cry3Aa12, Cry3Aa3, Cry3Bb1, Cry4Aa1, Cry4Ba1, Cry5Ba1, Cry7Ca1, and Cry8Ea1) [38,39,40,41,42,43,44,45,46,47,48], and some with modifications in the form of mutations (Cry1Ac4 and Cry1Da1) [49,50], have been determined by X-ray crystallography (Figure 2).

The tertiary structure of the N-terminal domain, called domain I, is a set of seven α-helices, among which the central α-helix is hydrophobic and surrounded by six amphipathic helices; this helical domain is responsible for membrane insertion and pore formation. Domain II consists of three antiparallel β sheets with exposed loop regions, and domain III is a β sheet [38,39,40,41,42,46]. The most exposed regions in the tertiary structure of the protein are domains II and III, which are involved in receptor binding [50]. 

The crystal protein consists of proteins called δ-endotoxin. The definition of Cry proteins is any parasporal protein of Bt that shows a toxic effect on an organism, verifiable using bioassays, or any protein that shows similarity to Cry proteins [51].

### 2.3. Action Mechanism of Cry as a Bioinsecticide

**Figure 3:**
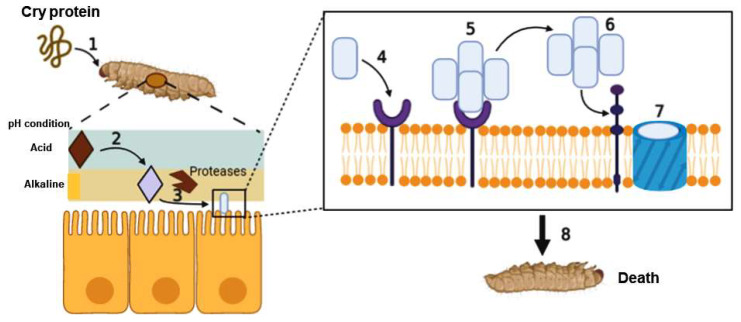
When an insect larva ingests *Bacillus thuringiensis* or the Cry protein present in bioinsecticides, it ingests crystals that may contain one or more Cry proteins (1). These crystals are solubilized due to the alkaline pH present in the midgut of the insect. After that, Cry proteins are released in the form of protoxins (inactive, active), which still lack toxic biological activity. Alkaline pH conditions ranging from 8 to 11 are found in lepidopteran and dipteran insects; some Cry proteins require neutral or slightly acidic pH conditions, which are present in coleopteran insects. Thus, Cry proteins are specific (2). Soluble Cry proteins cannot produce their effects until they are processed by intestinal proteases, generating active toxins, which requires the cleavage of peptides from both the N- and the C-termini (3). Subsequently, they bind to various membrane receptors of the cells of the insect’s intestine (4), form oligomers (5) until they locate and bind to a specific receptor, mainly cadherins, among others (see the text) (6), and lead to the formation of a pore (7), causing an osmotic imbalance, cell lysis, and finally, as a consequence, the death of the insect (8).

Crystal proteins have been widely used in genetically modified cultures; these transgenic cultures can produce Bt crystals, making them insect resistant. The two presentations of bioinsecticides, those that contain spores and toxic crystals or transgenic foods that express Cry proteins, have a similar mechanism [52]. Cry protein crystals need to be solubilized for their activation after they are ingested by an insect larva. The activation is mediated by the action of proteolytic enzymes, such as cathepsin G and chymotrypsin, which are located in the digestive system of the insect larva [53] and perform proteolytic processing at the amino terminus of the Cry protein [54]. Once the protein is activated it becomes the so-called δ-endotoxin; this soluble and partially truncated form of the protein is expressed in transgenic foods [52]. The δ-endotoxin binds to receptors located in the membrane of the epithelial cells of the intestine of the larva. Proteins such as cadherins [55], aminopeptidases, and alkaline peptidases [56] are recognized by the domains of δ-endotoxin and, recently, the binding of δ-endotoxin to other proteins, such as ATP-binding transporter proteins (ABC), has been demonstrated [57]. The cadherins that bind to Cry toxins in different orders of insects share a structure composed of four domains: an ectodomain (CE), a domain of the proximal extracellular membrane (MPED), a transmembrane domain (TM), and a cytoplasmic domain (CYTO). Several models have been proposed to explain the action mode of Cry proteins. One of them describes the process in several steps: solubilization of the crystal, processing of the protoxins, and binding to the receptor. The binding allows the oligomerization of δ-endotoxin in the membrane of intestinal cells, its insertion into the membrane, and aggregation, which results in the formation of a pore. This pore in the cell membrane causes an ionic imbalance (release of H^+^, K^+^, Na^+^, and Ca^2+^ ions) in the cell [58,59,60], which causes an increase in cAMP and, consequently, the activation of the apoptotic process known as cytolysis (Figure 3) [61]. Another model suggests that the toxin monomer can bind to a cadherin receptor and activate Mg^2+^-dependent signal transduction, a pathway that leads to cell death [62].

Some non-target insects and even some mammals, such as humans, are not sensitive to this bioinsecticide despite having the same receptors on the cell membrane; however, a difference in the structures of the receptors has been observed. Cadherin (Type IV) proteins in sensitive insects have eight or more cadherin domains, which facilitate the anchoring of δ-endotoxin, unlike the cadherins of resistant insects, which have few domains. For this reason, δ-endotoxin is specific because it binds to certain receptors in target insects [63,64]. Moreover, the proteins that allow proteolytic processing for the activation of the Cry protein are not present in the digestive system of resistant insects [54].

On the other hand, cadherins (type I) in humans have structural differences compared to insect cadherins; they principally have minor ectodomains (EC) and a few Ca^2+^ insertions, which confer foldability to the consecutive extracellular cadherin domains responsible for homophilic binding. This binding is also different in human cadherins because the EC1 domain of vertebrate cadherins contains a conserved tryptophan residue (W) inserted in the hydrophobic pocket, affecting homophilic binding [65]. Moreover, the identity between the cadherins of humans and those of insects (Diptera, Lepidoptera, and Coleoptera) is very low, ranging from 13% to 20% (Table 2). For these reasons, Cry proteins do not pose any potential toxicological risk to humans when they are ingested. 

## 3. Adjuvants Currently in Use

Traditional vaccines derived from toxins, or attenuated or inactivated live organisms are effective in inducing predominantly antibody-based immunity. Adjuvants (taken from Latin “adjuvare”, meaning “help”) are designed to improve the immune response to the vaccine [66]. Adjuvants were initially described as “substances used in combination with a specific antigen that produces a more effective immune response than the antigen alone”, thus encompassing a wide range of substances that can potentially function as an adjuvant [67].

Gaston Ramon, a French veterinarian, observed that the performance of antisera against tetanus and diphtheria in horses was higher in animals that developed an abscess at the injection site. By injecting starch, breadcrumbs, or tapioca, abscesses were sterilized at the site of injection and were, therefore, able to increase the production of antisera [68]. Subsequently, aluminum was used as an adjuvant in vaccines in 1932 [69]. Direct immunization with most antigens will lead to a poor immune response and rapid elimination of the antigen from the body. To avoid this, the antigen is first combined with an adjuvant. The formation of a stable emulsion between the antigen and the adjuvant allows the sustained presentation of the antigen to the immune system, and the removal of the antigen from the body is delayed. These agents can accelerate the development of the immune response against the antigen and ultimately lead to a stronger adaptive response than would otherwise be possible. The adjuvant should also provide the additional benefit of allowing the use of smaller amounts of the antigen [70]. Some important characteristics required of adjuvants are a high stability, a long useful life, biodegradability, and low cost to produce or obtain, and they should not induce immune responses against themselves but induce an appropriate immune response according to the requirements (immune response mediated by antibodies or cells) [66].

Many molecules have been considered for their use as adjuvants. Adjuvants have been classified into two groups: those that direct the antigen to antigen-presenting cells (APCs) and immunostimulatory types, which directly activate cells through specific receptors, producing inflammatory responses that amplify the innate immune response [69]. The ultimate aim of using an adjuvant is to activate the innate immune system to respond more quickly to stimuli and to enhance the specificity of the adaptive immune response [71]. Despite the multiple advantages of adjuvants for the potential action of a vaccine, only a few are currently used in licensed human vaccines (Table 3).

### 3.1. Benefits of Cry Relative to Other Adjuvants

Adjuvants that are currently used in human vaccines exhibit diverse immune response mechanisms. One of the well-characterized adjuvants is MF59. This adjuvant does not generate Th1-type immunity and, thus, its use in vaccines that are required to induce cell-mediated immunity for protection is not possible [84]. Another adjuvant is Lipopolysaccharide (LPS), which can induce the production of cytokines and chemokines in a variety of cells that can control the traffic and maturation of DC [85]. However, LPS has limitations that derive from its pyrogenicity and toxicity in animals and humans, so its use in vaccines as an adjuvant is rare.

Regarding the study of new adjuvants, cholera toxin (CT) from *Vibrio cholerae* and heat-labile toxin (LT) from *Escherichia coli* have been studied as mucosal adjuvants. Unfortunately, both are products derived from bacteria that are pathogenic to humans, preventing their use in vaccines despite being very good activators of the mucosal immune response. Furthermore, they have high production costs [86,87,88].

In this context, the emerging potential of Cry proteins as adjuvants is important because they are not toxic to vertebrates, including humans, and the cost of production is relatively low [26,89]. Moreover, their immunogenic and adjuvant capabilities, which are as potent as those of cholera toxin, have already been demonstrated [90]. 

Among the mechanisms of immune activation, the importance of specific antigen-enhancing adjuvants has been shown in various studies, which have demonstrated that their administration can activate both humoral and cellular immune responses [90,91,92,93]. The route of administration is also very important. Studies have shown that when administered orally, the Cry protein is highly immunogenic, requiring a very low dose, so the possible toxicity of this protein would be greatly diminished [5].

Because of these broad characteristics, Cry proteins are important proteins for the development of adjuvants for vaccine formulations against intra- or extracellular microorganisms. As previously discussed, adjuvants approved for vaccines are limited, and some of them only induce an immune response mechanism or are toxic in high doses.

### 3.2. An Overview of Cry as a Natural Adjuvant

A strategy used in the design of vaccines to enhance their immunogenicity includes the co-administration of adjuvants that stimulate and improve immunity. Cry proteins have been described as possible adjuvants for their resistance and stability in highly alkaline environments. The structural characteristics of proteins allow them to modulate the immune response and function as adjuvants. For this reason, several proteins have been studied in the context of therapeutic proteins [94]. Importantly, a wide variety of proteins have been used as adjuvants to immunize animals intranasally, which can stimulate a protective immune response in the lungs and upper respiratory tract and possibly at distant sites, such as the gastric and genital mucosa [95]. 

With respect to mucosal vaccines, when rhesus macaques were vaccinated intranasally with a trimeric gp41 protein coupled to virosomes to evaluate an HIV-1 vaccine, IgA antibodies increased in the genital tract, and immunization also prevented transmission of infection [96]. Studies have been conducted to identify proteins able to induce a mucosal immune response, which was initially realized by Guimares et al. when they demonstrated the immunogenicity of the Cry1Ab protein of *Bacillus thuringiensis*. When Cry1Ab was exposed to different pH values, the results showed that the protein was only slightly degraded at pH 2.0 and, most importantly, it maintained its immunoreactivity [97]. Subsequently, Cry proteins were used as adjuvants for their resistance and stability. This was demonstrated by immunizing BALB/c mice intranasally, which is an alkaline environment. After immunization, the mice were protected when they were infected by the bacterium *Streptococcus pneumoniae* [93] or the parasite *Naegleria fowleri* [98].

Because of these characteristics, several research groups have studied the use of Cry as a potential adjuvant [93,98,99,100]. The role of Cry proteins as vaccine adjuvants was initially observed with the Cry1Ac protein, which was administered by different routes, including intragastric, intraperitoneal (i.p.), and intranasal immunization [92]. The last two are the most efficient due to their ability to induce isotypes of IgA and IgG antibodies in the murine model. These antibodies demonstrated protection in animal models when they were infected with the bacterium *Brucella abortus* or with parasites *Naegleria fowleri*, *Plasmodium chabaudi*, *Plasmodium berghei*, and *Taenia crassiceps* [92,98,101,102,103]. In the case of *B. abortus*, an intracellular bacterium, the use of Cry1Ac with the RB51 *B. abortus* strain conferred protection against an intranasal challenge with the virulent strain *B. abortus* 2308 in BALB/c mice. The results showed that the vaccine conferred immunoprotection, as evidenced by a decrease in the splenic bacterial load in immunized animals. The proliferation of cytotoxic TCD8+ cells increased the production of TNF-α and IFN-γ, and the generation of an IgG2a antibody response was also observed. These results indicate that the use of the Cry1Ac protein as a mucosal adjuvant via the intranasal route may be a promising strategy for developing a vaccine against brucellosis [102].

When the Cry1Ac protein was administered together with total extracts of the free-living amoeba *N. fowleri*, the immunized animals had 100% protection against the development of meningoencephalitis; however, when the animals were immunized with the Cry1Ac protein alone, only 60% of infected mice survived [98].

On the other hand, mice previously treated with Cry1Ac and infected with *P. chabaudi* (considered non-lethal) had 100% survival compared to mice previously treated with PBS, which demonstrated 80% survival. Furthermore, mice previously treated with Cry1Ac and subsequently infected with *P. berghei* (lethal parasite) survived longer (12 days) than control mice previously treated with PBS, which died on day 9 post-infection. Regarding the induced immune response, an increase in IFN-γ and TGF-β cytokines was demonstrated, in addition to an increase in the levels of IgG and IgM immunoglobulins, in animals treated and infected with two types of Plasmodium [103].

Conversely, when mice were immunized with the Cry1Ac protein and total lysates of *T. crassiceps*, only 40% protection was observed in the experimentally infected animal model, and mice immunized only with Cry1Ac did not survive [92,98,99,100,101]. These results demonstrate that Cry1Ac alone is not able to generate a specific immune response; however, when it is used in the company of a specific antigen, it potentiates the immune response and can function as an adjuvant. At present, the immunological mechanism underlying the effects of the Cry1Ac protein is not entirely understood, but in macrophages this protein can stimulate the overexpression of surface glycoproteins CD80 and CD86, which stimulate the secretion of TNF-α and IL-6 cytokines, allowing activation of an immune response that promotes the survival of animal models against some experimental infections [104].

Another protein used as a potential adjuvant is Cry1Ab (a protein with 86% homology at the amino acid level with Cry1Ac). When administered intranasally, this protein was not able to generate serum and mucosal IgG antibody responses. It was only able to elicit a low level of IgM and SIgA. In contrast, when Cry1Ab was administered by the intraperitoneal route, it was able to induce high levels of IgG and IgM antibodies, similar to the effects of Cry1Aa and Cry1Ac [105]. In another study, the administration of Cry1Ab (1 µg) to BALB/c mice by the i.p. route induced a mixed Th1-Th2 immune response. No evidence of allergenicity has been observed with the administration of Cry proteins [106], though the levels of leukotrienes, cytokines, and eosinophils have notably increased. However, another study conducted with the direct consumption of Bt corn with Cry1Ab protein showed that the inclusion of Cry1Ab in the diet did not affect the severity of asthma or allergic inflammation induced by ovalbumin [104].

**Figure 4:**
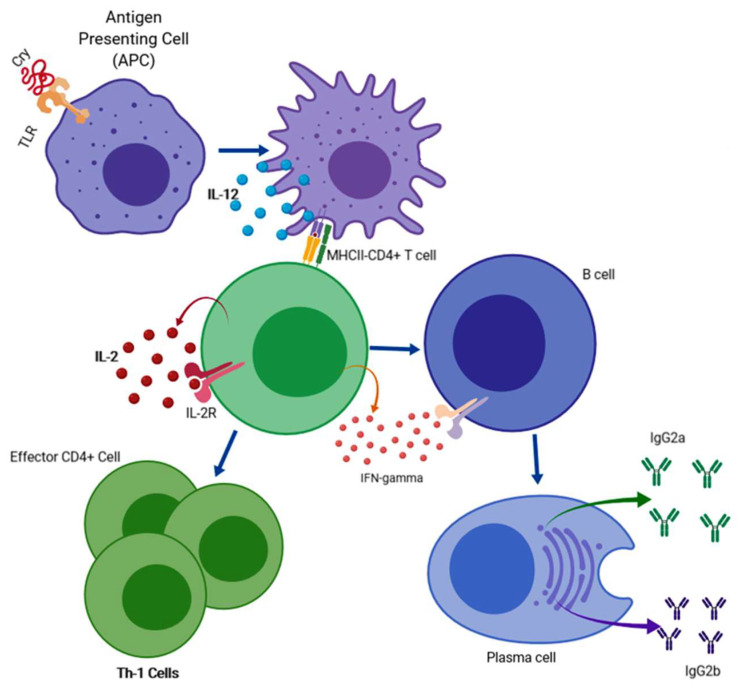
Based on the results obtained by several researchers, we propose the following putative mechanism of immune system activation by Cry proteins. Cry proteins could activate APCs through the recognition of Toll-like receptors and promote the production of IL-12, which leads to the activation of CD4+ T cells that can polarize towards a Th1 immune response. Th1 cells secrete cytokines such as IL-2 and IFN-gamma, leading to an increase in cell-mediated immunity. Furthermore, these activated CD4+ T cells can promote the activation of B cells, which then differentiate to form plasma cells that produce IgG immunoglobulins (IgG2a and IgG2b). The figure was created using BioRender.com (accessed on 18 September 2021).

Based on the previously described research, the possible immunological mechanism of Cry proteins may be the following: Cry proteins may be recognized by a Toll-like receptor located on the surface of antigen-presenting cells such as macrophages; many current adjuvants are recognized by various members of the Toll-like receptor (TLR) family [107]. These activated macrophages can secrete the IL-8 cytokine into the medium, which will recruit inflammatory cells such as neutrophils and immunological B and T lymphocytes. Once the immunodominant epitopes are exposed in the major histocompatibility complex type 1, they will be recognized by the TCR receptor of CD4+ lymphocytes, which will secrete cytokines such as IL-6, IL-8, TNF-α, and INF-γ. This last cytokine will be recognized by the INF-γ receptor exposed on B lymphocytes. Once the cytokine binds to the receptor, a series of signaling pathways will be activated inside the lymphocytes, which will induce the secretion of IgG2a and IgG2b antibodies (Figure 4).

The profile of cytokines and immunoglobulins activated by Cry proteins is the Th1 type; therefore, CD4+ T lymphocytes that differentiate into a subpopulation of T helper 1 cells could also activate this immune response. This type of response has been described as ideal for the elimination of a large variety of bacteria and parasites, principally intracellular. For this reason, a promising approach is to generate fused proteins by exploring Cry proteins together with any important protein of a pathogenic microorganism to use it as an adjuvant and potentiate a suitable immune response.

Finally, the activity of Cry proteins as bioinsecticides has been widely studied [51]; however, their other activities, such as their potential adjuvant function, have been inadequately explored. Although some experiments have confirmed their potential action as immunopotentiators [92,99,101], there are many Cry proteins that have not been evaluated yet. Furthermore, in different studies, the lack of toxicity of these proteins has been demonstrated in mammals, especially humans, and it has even been shown that these proteins can generate an immune response mediated by antibodies [108]. However, it is crucial to determine the mechanism by which Cry proteins are activated to induce an immune response, as well as to evaluate the possible risks that they could pose in the short and long term, such as allergies or other immune alterations. Therefore, we conclude that the use of Cry proteins as natural adjuvants is an interesting opportunity for new biotechnological processes and vaccine development.

## 4. Conclusions

To protect the health of the population against emerging and re-emerging infectious agents, the development of effective strategies is crucial, including vaccination. The current worldwide pandemic caused by the SARS-CoV-2 virus led to the rapid development of effective vaccines, which was possible because their platforms (adenovirus and mRNA) and adjuvants had been previously shown to work successfully. Requirements for adjuvants include low-cost components, a high production capacity, safety in humans, and the adequate potentiation of the immune response to the vaccine antigen. Some experimental studies have shown that Cry proteins from *Bacillus thuringiensis* are among such adjuvants. Therefore, in the coming years, it is important to study Cry proteins as adjuvants and begin to use them to promote the development of vaccines against new and old infectious agents.

## Figures and Tables

**Table 1:** Agricultural pests controlled by Cry toxins.

Cry Protein	Pests Controlled
Cry1A, Cry2A, Cry3A, Cry14A	Coleoptera
Cry1Ac, Cry2A	Lepidoptera
Cry1A, Cry2A, Cry4A, Cry10A	Diptera
Cry2A, Cry3A, Cry11A	Hemiptera
Cry3A, Cry5A, Cry22A	Hymenoptera

**Table 2:** Percentage of identity between human and insect cadherins.

Human Cadherin () ^1^	Insect Cadherin ^1^	Identity %
Type I (EAW83244.1)	*Drosophila melanogaster* (ACD79974.1) *Manduca sexta* (AAM21151.1) *Anopheles gambiae* (AGN95449.1) *Pieris rapae* (XP_022120264.1) *Bombyx mori* (XP_ 012545103.1) *Tribolium castaneum* (EEZ99177.2) *Aedes aegypti* (XP_021693027.1)	14.94 14.30 13.51 18.71 20.45 20.30 19.63
E-cadherin (CAA78353.1)	*Drosophila melanogaster* (ACD79974.1) *Manduca sexta* (AAM21151.1) *Anopheles gambiae* (AGN95449.1) *Pieris rapae* (XP_022120264.1) *Bombyx mori* (XP_ 012545103.1) *Tribolium castaneum* (EEZ99177.2) *Aedes aegypti* (XP_021693027.1)	14.42 14.03 13.29 18.43 20.51 20.14 19.74

^1^ Parentheses contain GenBank accession numbers of the respective amino acid sequences for the cadherins used in the table.

**Table 3:** Adjuvants used in human vaccines.

Adjuvant Composition	Immune Mechanism	Use in Vaccines	References
Aluminum salts (alum salts, aluminum hydroxide, aluminum phosphate, and aluminum sulfate phosphate)	Activation of the Nalp3/NLRP3 complex, leading to a considerable increase in IL-1β and IL-8. An increase in the chemokines CCL2, CCL3, and CCL4, which activate macrophages, has also been observed.	Diphtheria, pertussis, tetanus, hepatitis A and B viruses, meningococci, and human papillomavirus (HPV)	[69,72,73,74]
MF59 (combination of squalene, Polysorbate 80, and Span 85)	Directly induces the arrival of inflammatory cells, such as macrophages, and promotes the secretion of chemokines such as CCL4, CCL5, and CCL12. It can also activate Th1-type cytokines.	Simplex herpes virus, human immunodeficiency, and seasonal influenza	[72,75]
ASO3 (combination of squalene, vitamin E, and polysorbate)	Induces the arrival of macrophages, which secrete cytokines such as IL-6.	H5N1 and H1N1 influenza vaccines COVID-19 ^1^	[76,77] [78]
ASO4 (combination of monophosphoryl lipid A and aluminum salts)	TLR4-expressing cells in the muscle, such as resident or recruited dendritic cells or monocytes, are activated and induce the rapid recruitment and activation of monocytes and dendritic cells.	Hepatitis B virus (HBV) and HPV	[79,80,81]
Lipopolysaccharide (LPS)	Interacts directly on the TLR4 receptor, which can induce the secretion of cytokines TNF-α and IL-6 as well as various chemokines.	HPV and HBV	[82,83]

^1^ To be approved at the end of 2021.

## Data Availability

Not applicable.
